# Local Contrast-Based Pixel Ordering for Exact Histogram Specification

**DOI:** 10.3390/jimaging8090247

**Published:** 2022-09-10

**Authors:** Kohei Inoue, Naoki Ono, Kenji Hara

**Affiliations:** Department of Media Design, Faculty of Design, Kyushu University, 4-9-1 Shiobaru, Minamiku, Fukuoka 815-8540, Japan

**Keywords:** exact histogram equalization, exact histogram specification, Gaussian filter, contrast enhancement, pixel ordering

## Abstract

Histogram equalization is one of the basic image processing tasks for contrast enhancement, and its generalized version is histogram specification, which accepts arbitrary shapes of target histograms including uniform distributions for histogram equalization. It is well known that strictly ordered pixels in an image can be voted to any target histogram to achieve exact histogram specification. This paper proposes a method for ordering pixels in an image on the basis of the local contrast of each pixel, where a Gaussian filter without approximation is used to avoid the duplication of pixel values that disturbs the strict pixel ordering. The main idea of the proposed method is that the problem of pixel ordering is divided into small subproblems which can be solved separately, and then the results are merged into one sequence of all ordered pixels. Moreover, the proposed method is extended from grayscale images to color ones in a consistent manner. Experimental results show that the state-of-the-art histogram specification method occasionally produces false patterns, which are alleviated by the proposed method. Those results demonstrate the effectiveness of the proposed method for exact histogram specification.

## 1. Introduction

Histograms represent the distribution of pixel values in images, and can offer various image statistics [1] to us, i.e., histograms have useful information for image processing applications such as image enhancement, compression, and segmentation [2,3,4]. Therefore, histograms are the basis for numerous spatial domain processing techniques [5], which include intensity transformation as a special case where the smallest neighborhood of size 1×1 is used. Histogram equalization, which theoretically transforms an input image into the corresponding output image having a uniform histogram, is one of the intensity transformation techniques for contrast enhancement [5]. More generally, the method for generating an output image that has a specified target histogram is called histogram specification or histogram matching [5], in which histogram equalization is included as a special case [6]. It is pointed out that histogram equalization may cause over enhancement [7]. In such situations, histogram specification will be a good candidate for substitute methods because it accepts any shape of histograms. In this paper, although we would like to use a Gaussian distribution constantly for the target histogram in histogram specification to simplify our explanation, other choices such as bimodal or multimodal distribution may work well.

For digital images that are composed of pixels with quantized values, it is usual that the number of pixels in an image is greater than the number of possible quantized pixel values. Therefore, there are a large number of pixels that share the same quantized pixel value in a digital image, which means that exact histogram specification for digital images is an ill-posed problem [8]. For the effective utilization of all possible quantized pixel values and the suppression of the occurrence of false contours, a number of research works have been conducted for more than four decades since Hall published his work on discrete distribution transformation [9]. In addition to these technical needs, it is our genuine curiosity to make and see exactly histogram-equalized or specified images because the solution of the ill-posed problem is not unique. A promising approach to exact histogram specification is to obtain a meaningful strict ordering of all pixels in an image. Once we obtain the ordering of pixels, we can assign a limited number of pixel values to the ordered pixels so that the resultant histogram coincides with a specified target histogram.

The simplest way of pixel ordering is random ordering [10]. However, the random ordering is not a meaningful ordering. Coltuc et al. [11] described the process of exact histogram specification when a strict ordering is given, and proposed an ordering method, which is called the local mean method (LM), based on a filter bank of multiple moving average filters. Wan and Shi [12] proposed a wavelet-based exact pixel ordering algorithm (WA) for both exact histogram specification and image enhancement, which takes into account not only local mean intensity values, but also local edge information. Nikolova and Steidl [13] proposed a fast ordering algorithm for exact histogram specification based on a variational approach, which is equivalent to an iterative nonlinear filtering, and demonstrated that their variational ordering method outperforms both LM [11] and WA [12]. They also applied their algorithm to hue and range preserving enhancement of color images [14], where the histogram of an intensity image is exactly equalized or specified, and then an affine transform is applied to each color in a hue and range preserving manner.

In this paper, we propose a local contrast-based pixel ordering method for exact histogram specification, which computes the local contrast of each pixel by using a Gaussian filter [15] without approximation, and compare the proposed method with the state-of-the-art method by Nikolova and Steidl [13]. Furthermore, the proposed method for grayscale images is extended to that for color images in a consistent manner. Experimental results demonstrate that Nikolova and Steidl’s method can produce false patterns in flat areas of images, which are alleviated by the proposed method. For color images, it is experimentally shown that the proposed method outputs exactly histogram-equalized or specified images with closer hue to original images than that obtained by a separable method that utilizes Nikolova and Steidl’s method for exact histogram equalization and specification of each color channel. These results demonstrate the effectiveness of the proposed method compared with the state-of-the-art method.

The rest of this paper is organized as follows: Section 2 provides a brief review of some related works. Section 3 briefly summarizes conventional histogram equalization. Section 4 summarizes conventional histogram specification and Nikolova and Steidl’s exact histogram specification [13]. Section 5 proposes a local contrast-based exact histogram specification method, which is extended to that for color images. Section 6 shows experimental results, where the proposed method is compared with the state-of-the-art methods by Nikolova and Steidl [13] and Ramos et al. [16]. Finally, Section 7 concludes this paper.

## 2. Related Work

In this section, we review recent related works on histogram equalization and specification. Recently, Pallavi et al. [17] reviewed state-of-the-art techniques for image enhancement, in which the techniques of histogram equalization are also included such as contrast limited adaptive histogram equalization (CLAHE) [18] and brightness preserving bi-histogram equalization (BBHE) [19]. Trongtirakul and Agaian [20] proposed a weighted histogram equalization using entropy of probability density function, which outperformed related methods including a low-rank regularized retinex model (LR3M) [21]. More recently, Zhang et al. [22] proposed an unsupervised low-light image enhancement method via a histogram equalization prior (HEP) based on the observation that the feature maps of histogram equalization enhanced image and the ground truth are similar.

Hussain et al. [23] proposed a locally transformed histogram-based technique for dark image enhancement, which does not get affected from the over-enhancement problem. Balado [24] examined an optimum exact histogram specification problem and the inverse problem, and presented their closed-form performance analyses. Ramos et al. [16] proposed two algorithms for histogram specification and quantile transformation of data without local information. As one of the state-of-the-art algorithms for histogram specification, we implemented the first algorithm of Ramos et al. in this paper as described below. However, their algorithm does not produce an output image having the histogram being exactly identical to the target one.

The above review of recent related works draws a conclusion that Nikolova and Steidl’s algorithm [13] is still one of the state-of-the-art algorithms for exact histogram specification.

## 3. Histogram Equalization

Let $F=[f_{ij}]$ be a grayscale image for i=1,2,…,m and j=1,2,…,n, where $f_{ij}\in \left\{0,1,\ldots ,L\right\}$ denotes the pixel value at the position (i,j) on the image plane with *m* rows and *n* columns. The maximum pixel value *L* is typically given by $L=2^{8}-1=255$ in an 8-bit grayscale. Then, the histogram of the pixel values in *F* is given by $h=[h_{0},h_{1},\ldots ,h_{L}]$ whose *k*th element is computed by

(1) $$\begin{array}{c} h_{k}=\sum_{i=1}^{m}\sum_{j=1}^{n}\delta_{k,f_{ij}} \end{array}$$

for k=0,1,…,L, where $\delta_{k,f_{ij}}$ denotes the Kronecker delta function defined by $\delta_{k,f_{ij}}=0$ for $k\neq f_{ij}$, and 1 for $k=f_{ij}$. Note that the histogram h is not normalized, i.e., $\sum_{k=0}^{L}h_{k}=mn\neq 1$. The corresponding cumulative histogram is given by $H=[H_{0},H_{1},\ldots ,H_{L}]$ whose *l*th element is defined by

(2) $$\begin{array}{c} H_{l}=\sum_{k=0}^{l}h_{k} \end{array}$$

for l=0,1,…,L, and recursively computed by $H_{l}=H_{l-1}+h_{l}$ for l=1,2,…,L with $H_{0}=h_{0}$. Histogram equalization converts a pixel value $f_{ij}$ into

(3) $$\begin{array}{c} f_{ij}^{HE}=\phi_{h}^{HE}\left(f_{ij}\right):=\mathrm{round}\left(L\frac{H_{f_{ij}}-H_{f_{\min}}}{H_{f_{\max}}-H_{f_{\min}}}\right), \end{array}$$

where $f_{\min}=\min_{i,j}\left\{f_{ij}\right\},\;f_{\max}=\max_{i,j}\left\{f_{ij}\right\}$, and the ‘round’ operator rounds a given argument toward the nearest integer, and $\phi_{h}^{HE}$ denotes the function of converting pixel values in histogram equalization.

**Figure 1:**
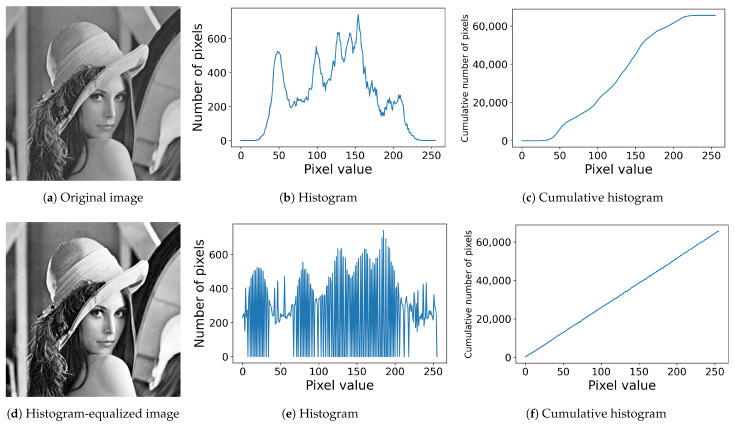
Histogram equalization: (**a**) an original image with 256×256 pixels; (**b**) the histogram of the original image; (**c**) the cumulative histogram of the original image; (**d**) the histogram-equalized image; (**e**) the histogram of the image in (**d**); (**f**) the cumulative histogram of the image in (**d**).

Figure 1 shows an example of histogram equalization, where an original image in Figure 1a is converted into the image in Figure 1d, and their histograms and cumulative ones are shown in Figure 1b,c and Figure 1e,f, respectively, where the horizontal axis of each graphic denotes the pixel value ranging from 0 to 255, and the vertical axis denotes the (cumulative) number of pixels for each (cumulative) histogram. As shown in Figure 1e, the resultant histogram is not equalized actually, but the cumulative one is linearized as shown in Figure 1f. That is, to be more precise, the conventional histogram equalization is a cumulative histogram linearization.

## 4. Histogram Specification

In this section, we summarize conventional histogram specification, which is not an exact method, and then briefly summarize the state-of-the-art method for exact histogram specification by Nikolova and Steidl [13]. After that, we propose a local contrast-based method for exact histogram specification, whose objects to be processed are extended from grayscale images to color images.

### 4.1. Conventional Histogram Specification

Let $\hat{h}=\left[{\hat{h}}_{0},{\hat{h}}_{1},\ldots ,{\hat{h}}_{L}\right]$ be a target histogram into which we want to convert the histogram h of the original image *F*, where we assume that $\sum_{k=0}^{L}{\hat{h}}_{k}=\sum_{k=0}^{L}h_{k}$ for convenience. Then, we can compute the cumulative histogram $\hat{H}=\left[{\hat{H}}_{0},{\hat{H}}_{1},\ldots ,{\hat{H}}_{L}\right]$ from $\hat{h}$ in the same manner as above. Theoretically, we can describe the relationship between the target histogram $\hat{h}$ and the original one h as

(4) $$\begin{array}{c} f_{ij}^{HS}={\left(\phi_{\hat{h}}^{HE}\right)}^{-1}\left(f_{ij}^{HE}\right)={\left(\phi_{\hat{h}}^{HE}\right)}^{-1}\left(\phi_{h}^{HE}\left(f_{ij}\right)\right), \end{array}$$

where $f_{ij}^{HS}$ denotes the output pixel value of histogram specification corresponding to an input pixel value $f_{ij}$, and ${\left(\phi_{\hat{h}}^{HE}\right)}^{-1}$ denotes the inverse function of $\phi_{\hat{h}}^{HE}$ which equalizes the target histogram $\hat{h}$. However, in the digital condition, the functions $\phi_{h}^{HE}$ and $\phi_{\hat{h}}^{HE}$ become staircase functions, which are not bijective. Therefore, they have no inverse functions.

A prescription for this situation may be the use of linear interpolation as follows: Assume that $\phi_{\hat{h}}^{HE}\left(l-1\right)<\phi_{h}^{HE}\left(f_{ij}\right)\leq \phi_{\hat{h}}^{HE}\left(l\right)$ for l∈{0,1,…,L}, where we define that $\phi_{\hat{h}}^{HE}\left(-1\right)=0$. Then, we can determine the output pixel value of histogram specification by

(5) $$\begin{array}{c} f_{ij}^{HS}\approx \mathrm{round}\left(\frac{\left[\phi_{\hat{h}}^{HE}\left(l\right)-\phi_{h}^{HE}\left(f_{ij}\right)\right]\max\left\{l-1,\;0\right\}+\left[\phi_{h}^{HE}\left(f_{ij}\right)-\phi_{\hat{h}}^{HE}\left(l-1\right)\right]l}{\phi_{\hat{h}}^{HE}\left(l\right)-\phi_{\hat{h}}^{HE}\left(l-1\right)}\right) \end{array}$$

corresponding to an input pixel value $f_{ij}$.

**Figure 2:**
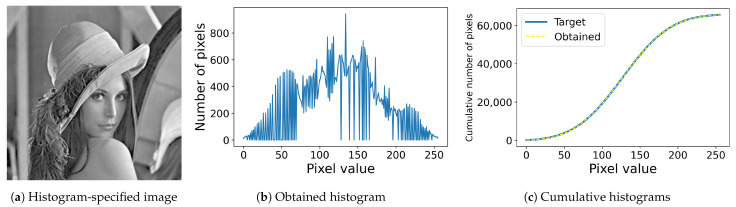
Histogram specification: (**a**) histogram-specified image computed from the original image in Figure 1a with a Gaussian target histogram; (**b**) the histogram of the image (**a**); (**c**) the target (blue solid line) and obtained (yellow dashed line) cumulative histograms.

Figure 2 shows an example of histogram specification, where the resultant image in Figure 2a is obtained with a Gaussian target histogram with mean 127.5 and standard deviation 50 from the original image in Figure 1a. Figure 2b shows the obtained histogram, which is not Gaussian, but the cumulative one fits into the target cumulative histogram as shown in Figure 2c, where a blue solid line denotes the target one, and the yellow dashed line denotes the obtained one. That is, to be more precise, the conventional histogram specification is a cumulative histogram specification.

### 4.2. Nikolova and Steidl’s Method

**Figure 3:**
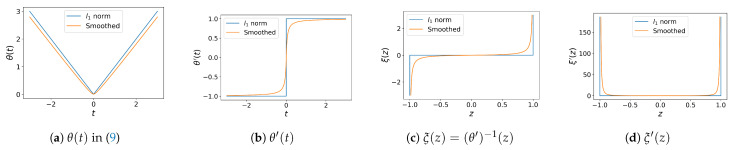
Nikolova and Steidl’s smooth approximation of $l_{1}$ norm: (**a**) $l_{1}$ norm denoted by a blue line is smoothly approximated by θ(t) denoted by an orange line; (**b**) the derivatives of $l_{1}$ norm and θ(t); (**c**) the inverse functions of $l_{1}$ norm and $\theta^{\prime }\left(t\right)$, which is denoted by ξ(z); (**d**) the derivatives of the inverse functions.

In this subsection, we briefly summarize Nikolova and Steidl’s fast ordering algorithm for exact histogram specification [13]. They proposed a fixed point algorithm that attains the minimizer of fully smoothed $l_{1}$-TV (total variation) functional as follows:

(6) $$\begin{array}{c} J\left(u,f\right):=\sum_{\iota =1}^{mn}\theta \left(u_{\iota }-f_{\iota }\right)+\beta \sum_{\kappa =1}^{r}\theta \left({\left(Gu\right)}_{\kappa }\right), \end{array}$$

where β>0, $f={\left[f_{1},f_{2},\ldots ,f_{mn}\right]}^{T}=\mathrm{vec}\left(F\right)={\left[f_{11},f_{21},\ldots ,f_{m-1,n},f_{mn}\right]}^{T}$ is the vectorization [25] of *F* where the superscript *T* denotes the matrix transpose [26], ${\left(Gu\right)}_{\kappa }$ denotes the κth component of $Gu\in R^{r}$ with r=2mn−m−n, where R denotes the set of real numbers, and *G* is a forward difference operator given by

(7) $$\begin{array}{c} G:=\left(\begin{array}{c} I_{n}⊗D_{m}\\ D_{n}⊗I_{m} \end{array}\right)\in R^{r,mn}, \end{array}$$

where ⊗ denotes the Kronecker product, $I_{n}$ is the n×n identity matrix, $D_{n}$ denotes the forward difference matrix defined by

(8) $$\begin{array}{c} D_{n}:=\left(\begin{array}{cccccc} -1 & 1 & \; & & \; & \\ \; & -1 & 1 & \; & & \\ \; & \; & & \ddots & \; & \\ \; & \; & & \; & -1 & 1 \end{array}\right)\in R^{n-1,n}, \end{array}$$

and the function θ is defined by

(9) $$\begin{array}{c} \theta \left(t\right):=\left|t\right|-\alpha \log\left(1+\frac{\left|t\right|}{\alpha }\right) \end{array}$$

with a constant α>0, which is a smooth approximation of $l_{1}$ norm, and has the derivative $z=\theta^{\prime }\left(t\right)=\frac{t}{\alpha +|t|}$. The inverse function of $\theta^{\prime }$ is defined by $\xi \left(z\right):={\left(\theta^{\prime }\right)}^{-1}\left(z\right)=\frac{\alpha z}{1-|z|}$, whose derivative is given by $\xi^{\prime }\left(z\right)=\frac{\alpha }{{\left(1-|z|\right)}^{2}}$. These functions are illustrated in Figure 3, where blue lines denote the functions for $l_{1}$ norm, and orange lines denote their smooth approximations.

From $\frac{\partial J}{\partial u_{\iota }}=\theta^{\prime }\left(u_{\iota }-f_{\iota }\right)+\beta \sum_{\kappa =1}^{r}\theta^{\prime }\left({\left(Gu\right)}_{\kappa }\right)G_{\kappa ,\iota }=0$ where $G_{\kappa ,\iota }$ means the (κ,ι)th element of *G*, we have $u_{\iota }=f_{\iota }-\xi \left(\beta \sum_{\kappa =1}^{r}\theta^{\prime }\left({\left(Gu\right)}_{\kappa }\right)G_{\kappa ,\iota }\right)$ for ι=1,2,…,mn, which are combined into a vector equation as $u=f-\xi (\beta G^{T}\theta^{\prime }\left(Gu\right))$, where the functions $\theta^{\prime }$ and ξ are applied to each element of their arguments. This is a fixed point equation for u which gives rise to a fixed point algorithm for minimizing J(u,f) as described in Algorithm 1.
**Algorithm 1** Fully smoothed $l_{1}$-TV minimization algorithm [13]**Require:** a vectorized image $f={\left[f_{1},f_{2},\ldots ,f_{mn}\right]}^{T}$, parameters β>0 and α>0 used in functions ξ and $\theta^{\prime }$, the number of iterations T>0**Ensure:** 
the minimizer $u^{\left(T\right)}$ of a fully smoothed $l_{1}$-TV functional J(u,f) in (6)  1:Initialize the variable u as $u^{\left(0\right)}\leftarrow f$  2:**for** t←1,2,…,T**do**  3:    $u^{\left(t\right)}\leftarrow f-\xi \left(\beta G^{T}\theta^{\prime }\left(Gu^{\left(t-1\right)}\right)\right)$  4:**end for**

**Figure 4:**
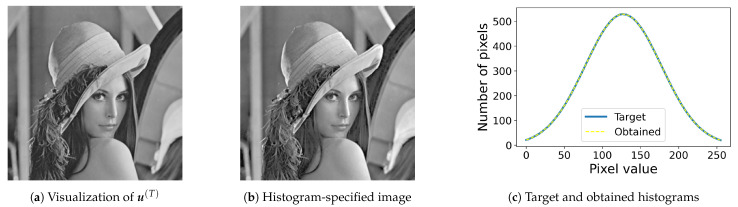
Nikolova and Steidl’s exact histogram specification: (**a**) the output $u^{\left(T\right)}$ of Algorithm 1 is visualized as an 8-bit grayscale image; (**b**) the output image; (**c**) Gaussian target (blue solid line) and obtained (yellow dashed line) histograms.

In this algorithm, we set α=0.05,β=0.1 and T=5 according to Nikolova and Steidl [13]. The obtained image from $u^{\left(T\right)}$ with the input image f in Figure 1a is shown in Figure 4a. This procedure for computing $u^{\left(T\right)}$ from f can be viewed as a nonlinear filter which reduces the quantization noise in f slightly. Therefore, the image in Figure 4a is very close to that in Figure 1a. The pixel values in $u^{\left(T\right)}$ can be ordered in a strict way with a high probability [8].

Assume that the pixel values in $u^{\left(T\right)}={\left[u_{1}^{\left(T\right)},u_{2}^{\left(T\right)},\ldots ,u_{mn}^{\left(T\right)}\right]}^{T}$ are ordered in ascending order: $u_{\lambda_{1}}^{\left(T\right)}<u_{\lambda_{2}}^{\left(T\right)}<\cdots <u_{\lambda_{mn}}^{\left(T\right)}$ where $\lambda_{\iota }$ for ι=1,2,…,mn denote the ordered indices, and the cumulative histogram $\hat{H}=\left[{\hat{H}}_{0},{\hat{H}}_{1},\ldots ,{\hat{H}}_{L}\right]$ is computed from a given target histogram $\hat{h}$. Then, if ${\hat{H}}_{l-1}<\iota \leq {\hat{H}}_{l}$ with ${\hat{H}}_{-1}=0$ for an index ι and a pixel value *l*, then the corresponding output pixel value is given by $f_{i_{\iota },j_{\iota }}^{NS}=l$, where $\left(i_{\iota },j_{\iota }\right)$ denotes the pixel position corresponding to the ordered index $\lambda_{\iota }$, i.e., $\left(\lambda_{\iota }-1\right)=\left(j_{\iota }-1\right)m+\left(i_{\iota }-1\right)$ with $0\leq (i_{\iota }-1)<m$ is satisfied. This procedure is repeated for ι=1,2,…,mn to obtain Nikolova and Steidl’s output image $F^{NS}=\left[f_{ij}^{NS}\right]$. Figure 4b shows the output image with the same Gaussian target histogram as Figure 2, and Figure 4c shows the target and obtained histograms, where we can see that they have the same Gaussian shape, which demonstrates the exactness of Nikolova and Steidl’s method.

## 5. Proposed Method

In this section, we propose a local contrast-based pixel ordering method for exact histogram specification. We first describe the method for grayscale images, and then it is extended to that for color images. We also give a complexity analysis of the proposed method. The main idea of our approach is that the problem of pixel ordering can be divided into 256 subproblems for an 8-bit grayscale image. We solve the downsized subproblems separately to have 256 groups of ordered pixels, and then concatenate them to have a sequence of all ordered pixels.

### 5.1. Local Contrast-Based Exact Histogram Specification for Grayscale Images

As demonstrated above, we can transform the histogram of a digital image into the specified one, when all pixels in the image are ordered in a strict and faithful way [13]. In most cases, pixels in a digital image take a limited number of discrete values, e.g., 256 values are available for 8-bit grayscale images, which is smaller than the number of pixels in the image, e.g., m×n=256×256=65,536≫256 for the above image in Figure 1a. Therefore, many pixels share the same pixel value with each other in the image, and the ordering of the pixels with the same values becomes an ill-posed problem [8]. However, it is fortunate for us that we have a rough ordering of 256 groups of pixels in the image, which means that the entire pixel ordering problem can be divided into 256 subproblems that can be solved separately. In this subsection, we propose a method for ordering pixels in each group on the basis of the local contrast of each pixel, and then the separately ordered subgroups are finally merged into an entire pixel ordering.

The first step of the proposed method is Gaussian filtering without approximation, which means that the weighted mean of all pixels is outputted at each pixel, where the weights are given by a Gaussian function as follows:

(10) $$\begin{array}{c} f_{ij}^{GF}=\frac{\sum_{k=1}^{m}\sum_{l=1}^{n}\exp\left(-\frac{{\left(i-k\right)}^{2}+{\left(j-l\right)}^{2}}{2\sigma^{2}}\right)f_{kl}}{\sum_{k=1}^{m}\sum_{l=1}^{n}\exp\left(-\frac{{\left(i-k\right)}^{2}+{\left(j-l\right)}^{2}}{2\sigma^{2}}\right)}=\frac{\sum_{k=1}^{m}\exp\left(-\frac{{\left(i-k\right)}^{2}}{2\sigma^{2}}\right)\sum_{l=1}^{n}\exp\left(-\frac{{\left(j-l\right)}^{2}}{2\sigma^{2}}\right)f_{kl}}{\sum_{k=1}^{m}\exp\left(-\frac{{\left(i-k\right)}^{2}}{2\sigma^{2}}\right)\sum_{l=1}^{n}\exp\left(-\frac{{\left(j-l\right)}^{2}}{2\sigma^{2}}\right)} \end{array}$$

for i=1,2,…,m and j=1,2,…,n, where σ denotes the standard deviation positive constant. Let $F^{GF}=\left[f_{ij}^{GF}\right]$ be an m×n matrix which expresses the Gaussian-filtered image of *F*. Then, we can compute $F^{GF}$ by the following matrix operations. The numerator of (10) is computed by

(11) $$\begin{array}{c} N_{u}=G_{L}FG_{R}, \end{array}$$

where the left matrix $G_{L}$ is given by $G_{L}=\exp\left(-D_{L}⊙D_{L}/2\sigma^{2}\right)$ with

(12) $$\begin{array}{c} D_{L}=1_{m}\left[\begin{array}{cccc} 1 & 2 & \cdots & m \end{array}\right]+{\left[\begin{array}{cccc} 1 & 2 & \cdots & m \end{array}\right]}^{T}1_{m}^{T}-2{\left[\begin{array}{cccc} 1 & 2 & \cdots & m \end{array}\right]}^{T}\left[\begin{array}{cccc} 1 & 2 & \cdots & m \end{array}\right], \end{array}$$

where ⊙ denotes the Hadamard product or elementwise product of matrices, and $1_{m}$ denotes the *m*-dimensional column vector of ones. Similarly, the right matrix $G_{R}$ in (11) is given by $G_{R}=\exp\left(-D_{R}⊙D_{R}/2\sigma^{2}\right)$ with

(13) $$\begin{array}{c} D_{R}=1_{n}\left[\begin{array}{cccc} 1 & 2 & \cdots & n \end{array}\right]+{\left[\begin{array}{cccc} 1 & 2 & \cdots & n \end{array}\right]}^{T}1_{n}^{T}-2{\left[\begin{array}{cccc} 1 & 2 & \cdots & n \end{array}\right]}^{T}\left[\begin{array}{cccc} 1 & 2 & \cdots & n \end{array}\right]. \end{array}$$

On the other hand, the denominator of (10) is computed by

(14) $$\begin{array}{c} D_{e}=G_{L}EG_{R}, \end{array}$$

where *E* denotes the m×n matrix of ones. Then, we have the Gaussian-filtered image by $F^{GF}=N_{u}⊘D_{e}$, where ⊘ denotes the Hadamard division or elementwise division of matrices.

Next, we define the local contrast (LC) of each pixel by $d_{ij}:=f_{ij}-f_{ij}^{GF}$ which is added to the corresponding pixel as an attribute, and divide the mn pixels in *F* into (L+1) groups as $S_{k}:=\left\{\left(f_{ij},d_{ij}\right)\;|\;f_{ij}=k\right\}$, where we have that $h_{k}=\left|S_{k}\right|$ for k=0,1,…,L, i.e., $h_{k}$ is the cardinality of $S_{k}$. Then, we sort the elements of $S_{k}$ in the ascending order of $d_{ij}$ for k=0,1,…,L, and concatenate them to have a sequence. As a result, we expect to have the entire pixel ordering $\mu_{\iota }\in \left\{1,2,\ldots ,mn\right\}$ for ι=1,2,…,mn, from which the corresponding pixel position $\left(i_{\iota },j_{\iota }\right)$ is given as the integer part $\left(j_{\iota }-1\right)$ and remainder $\left(i_{\iota }-1\right)$ of the division $(\mu_{\iota }-1)/m$, i.e., $\left(\mu_{\iota }-1\right)=\left(j_{\iota }-1\right)m+\left(i_{\iota }-1\right)$, and $\left\{d_{ij}\right\}$ is ordered as follows:

(15) $$\begin{array}{c} d_{i_{1},j_{1}}<\cdots <d_{i_{\iota },j_{\iota }}<\cdots <d_{i_{mn},j_{mn}}. \end{array}$$

For exact histogram specification, we would like to have a strict ordering as described in (15). That is, for different pixels with the same pixel value as $f_{ij}=f_{i^{\prime }j^{\prime }}$ with $i\neq i^{\prime }$ or $j\neq j^{\prime }$, it is expected that $d_{ij}\neq d_{i^{\prime }j^{\prime }}$ or $f_{ij}^{GF}\neq f_{i^{\prime }j^{\prime }}^{GF}$. For conventional Gaussian filters with finite kernel sizes such as 3×3,5×5 and 7×7 pixels, the lower bounds of the probability of $f_{ij}^{GF}=f_{i^{\prime }j^{\prime }}^{GF}$ are ${\left(\frac{1}{256}\right)}^{7\times 7}<{\left(\frac{1}{256}\right)}^{5\times 5}<{\left(\frac{1}{256}\right)}^{3\times 3}\approx 0$. However, some symmetric patterns may result in the case of $f_{ij}^{GF}=f_{i^{\prime }j^{\prime }}^{GF}$ accidentally. To reduce the probability of such cases, we adopt the Gaussian filter with sufficiently large kernel size as described in (10).

For an input image *F* with a target histogram $\hat{h}$ and its cumulative version $\hat{H}=\left[{\hat{H}}_{0},{\hat{H}}_{1},\ldots ,{\hat{H}}_{L}\right]$, the proposed method outputs a histogram-specified image $F^{LC}=\left[f_{ij}^{LC}\right]$, where each pixel value is given by $f_{i_{\iota },j_{\iota }}^{LC}=l$ for the index $\mu_{\iota }$ satisfying ${\hat{H}}_{l-1}<\iota \leq {\hat{H}}_{l}$ with ${\hat{H}}_{-1}=0$ as well as Nikolova and Steidl’s method summarized in Section 4.2. The proposed method is summarized in Algorithm 2.
**Algorithm 2** Local contrast-based exact histogram specification**Require:** 
a grayscale image $F=[f_{ij}]$, a positive constant σ and a target histogram $\hat{h}$**Ensure:** 
a histogram-specified image $F^{LC}=\left[f_{ij}^{LC}\right]$  1:Compute the Gaussian-filtered image $F^{GF}$ of *F* with a positive constant σ  2:Compute the local contrast $D=[d_{ij}]$ by $D=F-F^{GF}$  3:Make (L+1) groups $S_{k}=\left\{\left(f_{ij},d_{ij}\right)\;|\;f_{ij}=k\right\}$ for k=0,1,…,L  4:ι←1  5:**for** k←0,1,…,L **do**  6:      Sort the elements of $S_{k}$ in the ascending order of $d_{ij}$ as $d_{i_{1},j_{1}}<\cdots <d_{i_{u},j_{u}}<\cdots <d_{i_{|S_{k}|},j_{|S_{k}|}}$  7:      **for** $u\leftarrow 1,2,\ldots ,|S_{k}|$ **do**  8:            $\mu_{\iota }\leftarrow m\left(j_{u}-1\right)+i_{u}$  9:            ι←ι+110:       **end for**11:**end for**12:Compute the target cumulative histogram $\hat{H}=\left[{\hat{H}}_{0},{\hat{H}}_{1},\ldots ,{\hat{H}}_{L}\right]$ from $\hat{h}$13:**for** ι←1,2,…,mn **do**14:      Search for *l* satisfying ${\hat{H}}_{l-1}<\iota \leq {\hat{H}}_{l}$ with ${\hat{H}}_{-1}=0$15:      $f_{i_{\iota },j_{\iota }}^{LC}\leftarrow l$, where $\left(j_{\iota }-1\right)$ and $\left(i_{\iota }-1\right)$ are the integer part and remainder of $(\mu_{\iota }-1)/m$16:**end for**

Additionally, we can make an image whose pixel values indicate ordered numbers as $F^{I}=\left[f_{ij}^{I}\right]$, where each pixel value is given by $f_{i_{\iota },j_{\iota }}^{I}=\iota$ for ι=1,2,…,mn. For histogram equalization, the number of pixels to which the same pixel value is assigned is given by $\bar{h}=\left\lfloor mn/\left(L+1\right)\right\rfloor$, where ⌊x⌋ denotes the floor function that gives the largest integer less than or equal to *x* [27]. That is, we make a target histogram $\hat{h}=\left[{\hat{h}}_{k}\right]$ as ${\hat{h}}_{k}=\bar{h}$ for k=0,1,…,L. However, the truncation by the floor function may cause the shortage of total amount in the histogram: $\sum_{k=0}^{L}{\hat{h}}_{k}<mn$. To cover the shortage, if $Q=mn-\sum_{k=0}^{L}{\hat{h}}_{k}>0$, then we add 1 to ${\hat{h}}_{0},{\hat{h}}_{1},\ldots ,{\hat{h}}_{Q-1}$ to have $\sum_{k=0}^{L}{\hat{h}}_{k}=mn$. This modified histogram can be inputted to Algorithm 2 as a target histogram for exact histogram equalization.

**Figure 5:**
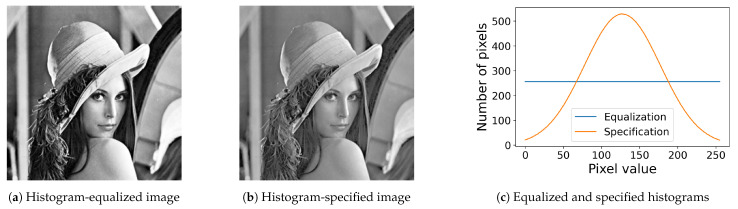
Exact histogram equalization and specification: (**a**) histogram-equalized image of the original image in Figure 1a; (**b**) the histogram-specified image with a Gaussian target histogram; (**c**) equalized (blue line) and specified (orange line) histograms.

Figure 5 shows the results of the proposed method, where Figure 5a,b show the results of the histogram equalization and specification with σ=50 for the original image in Figure 1a, respectively, and Figure 5c shows their histograms, where we can see that both exact histogram equalization and specification are achieved.

### 5.2. Extension to Color Images

In this subsection, we extend the above proposed method for grayscale images to color images. Let $C=[c_{ij}]$ be an RGB color image, where $c_{ij}=\left[c_{ij1},c_{ij2},c_{ij3}\right]$ denotes the RGB color vector of the (i,j)th pixel in *C*, and $c_{ijk}\in \left\{0,1,\ldots ,L\right\}$ for k=1,2 and 3. Then, we make a histogram into which all RGB values in *C* are united as follows:

(16) $$\begin{array}{c} h^{C}=\left[h_{0}^{C},\ldots ,h_{l}^{C},\ldots ,h_{L}^{C}\right],\;h_{l}^{C}=\sum_{i=1}^{m}\sum_{j=1}^{n}\sum_{k=1}^{3}\delta_{l,c_{ijk}}\;\mathrm{for}\;l=0,1,\ldots ,L. \end{array}$$

**Figure 6:**
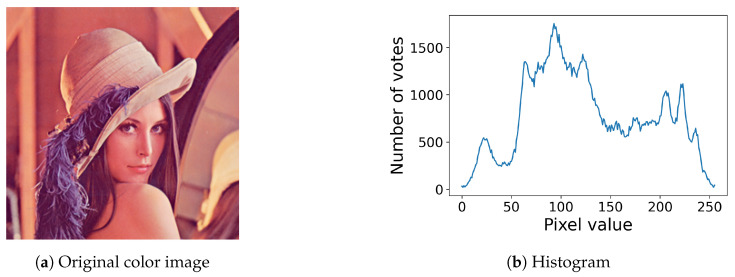
Color image and its histogram: (**a**) an RGB color image with 256×256 pixels; (**b**) the histogram of the color image (**a**).

For example, Figure 6 shows an RGB color image and its histogram defined in (16).

The first step of our exact histogram specification is Gaussian filtering applied to each color channel in *C*. Let $C^{GF}=\left[c_{ij}^{GF}\right]$ be the resultant image, where $c_{ij}^{GF}=\left[c_{ij1}^{GF},c_{ij2}^{GF},c_{ij3}^{GF}\right]$ denotes the RGB color vector of each pixel in $C^{GF}$. Then, we compute the LC as $D^{C}=\left[d_{ij}^{C}\right]=C-C^{GF}$ where $d_{ij}^{C}=\left[d_{ij1}^{C},d_{ij2}^{C},d_{ij3}^{C}\right]=c_{ij}-c_{ij}^{GF}$, each element of which is added to the corresponding element of the corresponding pixel as an attribute: $\left(c_{ijk},d_{ijk}^{C}\right)$ for i=1,2,…,m;j=1,2,…,n and k=1,2,3, and divide them into (L+1) groups as $S_{l}:=\left\{\left(c_{ijk},d_{ijk}^{C}\right)\;|\;c_{ijk}=l\right\}$, where we have that $h_{l}^{C}=\left|S_{l}\right|$ for l=0,1,…,L. Then, we sort the elements of $S_{l}$ in the ascending order of $d_{ijk}^{C}$ for l=0,1,…,L, and concatenate them to have a sequence. As a result, we expect to have the entire element ordering $\eta_{\iota }\in \left\{1,2,\ldots ,3mn\right\}$ for ι=1,2,…,3mn, each of which is connected with the corresponding pixel position $\left(i_{\iota },j_{\iota }\right)$ and channel number $k_{\iota }$ by satisfying

(17) $$\begin{array}{c} d_{i_{1},j_{1},k_{1}}^{C}<\cdots <d_{i_{\iota },j_{\iota },k_{\iota }}^{C}<\cdots <d_{i_{3mn},j_{3mn},k_{3mn}}^{C}. \end{array}$$

Let ${\hat{h}}^{C}$ be a target histogram with the cumulative one ${\hat{H}}^{C}=\left[{\hat{H}}_{0}^{C},{\hat{H}}_{1}^{C},\ldots ,{\hat{H}}_{L}^{C}\right]$. Then, the histogram-specified image $C^{LC}=\left[c_{ij}^{LC}\right]$ is given by $c_{ij}^{LC}=\left[c_{ij1}^{LC},c_{ij2}^{LC},c_{ij3}^{LC}\right]$ with $c_{i_{\iota },j_{\iota },k_{\iota }}^{LC}=l$ for the index $\eta_{\iota }$ satisfying ${\hat{H}}_{l-1}^{C}<\iota \leq {\hat{H}}_{l}^{C}$ with ${\hat{H}}_{-1}^{C}=0$. This procedure for color images is summarized in Algorithm 3.
**Algorithm 3** Local contrast-based exact histogram specification for color images**Require:** 
a color image $C=[c_{ij}]$, a positive constant σ and a target histogram ${\hat{h}}^{C}$**Ensure:** 
a histogram-specified image $C^{LC}=\left[c_{ij}^{LC}\right]$  1:Compute the Gaussian-filtered image $C^{GF}$ of *C* with a positive constant σ  2:Compute the local contrast $D^{C}=\left[d_{ij}^{C}\right]$ by $D^{C}=C-C^{GF}$ where $d_{ij}^{C}=\left[d_{ij1}^{C},d_{ij2}^{C},d_{ij3}^{C}\right]$  3:Make (L+1) groups $S_{l}=\left\{\left(c_{ijk},d_{ijk}^{C}\right)\;|\;c_{ijk}=l\right\}$ for l=0,1,…,L  4:ι←1  5:**for** l←0,1,…,L **do**  6:      Sort the elements of $S_{l}$ in the ascending order of $d_{ijk}^{C}$ as $d_{i_{1},j_{1},k_{1}}^{C}<\cdots <d_{i_{u},j_{u},k_{u}}^{C}<\cdots <d_{i_{|S_{l}|},j_{|S_{l}|},k_{|S_{l}|}}^{C}$  7:       **for** $u\leftarrow 1,2,\ldots ,|S_{l}|$ **do**  8:             $\eta_{\iota }\leftarrow mn\left(k_{u}-1\right)+m\left(j_{u}-1\right)+i_{u}$  9:             ι←ι+110:        **end for**11:**end for**12:Compute the target cumulative histogram $\hat{H}=\left[{\hat{H}}_{0},{\hat{H}}_{1},\ldots ,{\hat{H}}_{L}\right]$ from $\hat{h}$13:**for** ι←1,2,…,3mn **do**14:      Search for *l* satisfying ${\hat{H}}_{l-1}<\iota \leq {\hat{H}}_{l}$ with ${\hat{H}}_{-1}=0$15:      $c_{i_{\iota },j_{\iota },k_{\iota }}^{LC}\leftarrow l$, where $i_{\iota },\;j_{\iota }$ and $k_{\iota }$ are related to $\eta_{\iota }$ by $\eta_{\iota }=mn\left(k_{\iota }-1\right)+m\left(j_{\iota }-1\right)+i_{\iota }$16:**end for**

**Figure 7:**
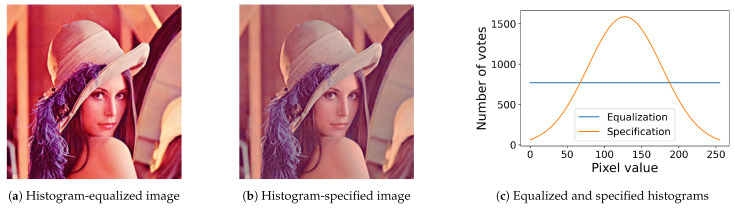
Exact histogram equalization and specification for a color image: (**a**) histogram-equalized image of the original color image in Figure 6a; (**b**) the histogram-specified image with a Gaussian target histogram; (**c**) equalized (blue line) and specified (orange line) histograms.

Figure 7 shows the results of the proposed method for color images, where Figure 7a,b show the results of the histogram equalization and specification with σ=50 for the original color image in Figure 6a, respectively, and Figure 7c shows their histograms, where we can also see that both exact histogram equalization and specification are achieved.

### 5.3. Complexity Analysis

In this subsection, we analyze the complexity of the proposed method in Algorithm 2. Let N=mn be the input size. The computation of Gaussian filtering in line 1 is described as matrix calculations. The time complexity of the numerator (11) is estimated as $mn^{2}+m^{2}n=mn\left(m+n\right)=N\left(m+n\right)\approx N^{3/2}$, which is the same as that of the denominator (14). The Hadamard division requires *N* elementwise divisions. Then, the order of time complexity of Gaussian filtering is $O(N^{3/2})$. The next main procedure is sort in line 6, which is repeated L+1 times. Assume that the cardinality $|S_{l}|$ is $\frac{N}{L+1}$ on average. Then, the time complexity of the sorting procedure is estimated as $\left(L+1\right)\times \frac{N}{L+1}\log\frac{N}{L+1}=N\left[\log N-\log\left(L+1\right)\right]$ from which we have O(NlogN). The time complexity of the remaining procedures is bounded by O(N). Consequently, the time complexity of the proposed method is given by $O(N^{3/2})$.

The space complexities of images $F,\;F^{GF},\;D$, and $F^{LC}$ are O(N), and that of (L+1) groups $S_{k}$ for k=0,1,…,L in line 3 is also O(N). Histogram arrays $\hat{h}$ and $\hat{H}$ require O(L+1). Selecting the largest one, we can see that the space complexity of the proposed method is O(N).

## 6. Experimental Results

**Figure 8:**
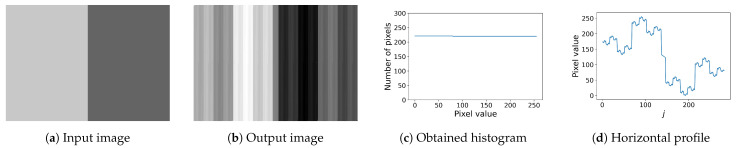
Nikolova and Steidl’s exact histogram equalization: (**a**) input image with 200×282 pixels, where the dark and bright areas have pixel values 100 and 200, respectively. (**b**) vertical stripes are generated in the output image; (**c**) the obtained histogram confirms that Nikolova and Steidl’s method exactly equalizes the histogram; (**d**) horizontal profile in (**b**) shows the fluctuation of pixel values, but it preserves the original ordering in (**a**).

In this section, we show experimental results in comparison with Nikolova and Steidl’s method [13]. First, we show the results with a synthetic input image shown in Figure 8a. Nikolova and Steidl’s method summarized in Section 4.2 outputs the image in Figure 8b, where we can see that vertical stripes appear in the flat areas in the original image. As a result, Nikolova and Steidl’s method achieves the equalized histogram shown in Figure 8c. Figure 8d shows the horizontal profile of the output image in Figure 8b, where the vertical and horizontal axes denote the pixel value and the column index *j*, respectively. Although the pixel values fluctuate with small steps, the pixel values on the left side are larger than that on the right side as well as the original image.

**Figure 9:**
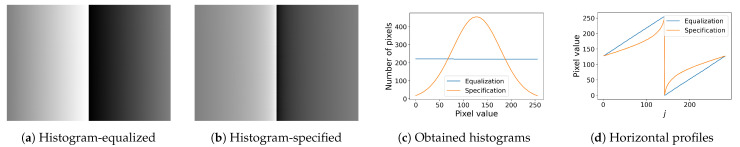
Exact histogram equalization and specification by the proposed method: (**a**) histogram-equalized image has the enhanced central edge; (**b**) histogram-specified image also has the enhanced edge; (**c**) histograms of the output images in (**a**,**b**) are equalized and specified to be Gaussian, respectively; (**d**) horizontal profiles in (**a**,**b**) have no fluctuation observed in Figure 8d.

Figure 9 shows the results of the proposed method described in Section 5, where Figure 9a,b show the output images of exact histogram equalization and specification with the Gaussian target histogram used in the previous section. Their histograms are shown in Figure 9c. The proposed method enhances the edge in the center of the input image in Figure 8a, and does not produce the pseudo-stripes observed in Figure 8b that are graphically confirmed by the horizontal profiles in Figure 9d.

**Figure 10:**
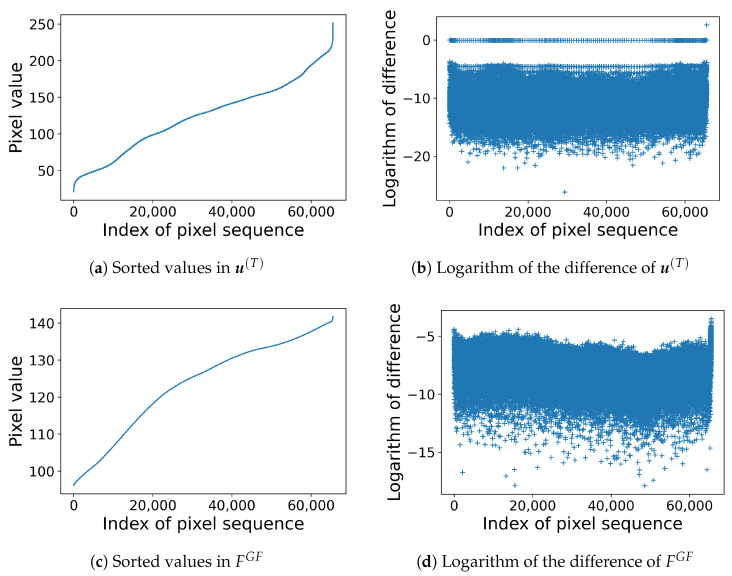
Comparison of filter outputs $u^{\left(T\right)}$ and $F^{GF}$: (**a**) The values in the output $u^{\left(T\right)}$ in Algorithm 1 visualized in Figure 4a are sorted in ascending order. (**b**) The values of $\log(u_{\lambda_{\iota +1}}^{\left(T\right)}-u_{\lambda_{\iota }}^{\left(T\right)})$ for ι=1,2,…,mn−1 are plotted. (**c**) The values of the output of the Gaussian filter in (10) with σ=50 are sorted in ascending order. (**d**) The logarithm of the difference of the sorted values in (**c**) are plotted.

Next, we compare the ability in strict ordering by Nikolova and Steidl’s and the proposed methods. Figure 10 compares the filter outputs used in the two methods with the input image in Figure 1a. Nikolova and Steidl’s filter in Algorithm 1 outputs $u^{\left(T\right)}$, the values in which are sorted in ascending order in Figure 10a where the vertical and horizontal axes denote the pixel value and index, respectively. The differences between the neighboring pixel values in Figure 10a are very small, and therefore, we take the logarithm of them as shown in Figure 10b. Similarly, we sort the values of the output of the Gaussian filter in (10) as shown in Figure 10c, and take the logarithm of the difference values in Figure 10c as shown in Figure 10d, where we can see that the minimum value is greater than that in Figure 10c, which is preferable for strict ordering because the larger the difference is, the clearer the ordering is. The minimum values of the differences without taking logarithm are $4.23\times 10^{-12}$ and $1.66\times 10^{-8}$ for Figure 10b,d, respectively.

**Figure 11:**
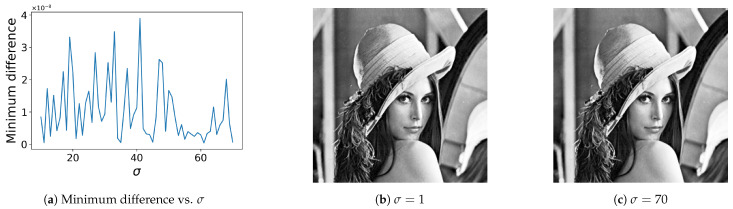
Insensitivity to parameter σ: (**a**) the minimum difference in the sorted values of $F^{GF}$ as a function of σ. The positive values mean that there are no pixels with the same pixel value in Gaussian-filtered image $F^{G}F$, from which we have a strict ordering; (**b**) histogram equalization with σ=1; (**c**) histogram equalization with σ=70.

The proposed method has a parameter σ in (10), the effect of which is investigated in Figure 11a, where the vertical and horizontal axes denote the minimum difference in the sorted output values of the Gaussian filter and the parameter σ, respectively, for the input image in Figure 1a. In this figure, although we cannot find clear tendency of the minimum difference as a function of σ, the obtained difference values are larger than that of $u^{\left(T\right)}$ given by Algorithm 1 that exemplifies the insensitivity of the proposed method to σ. Figure 11b,c show the results of histogram equalization with σ=1 and 70, respectively. These images demonstrate that the proposed method is insensitive to the values of parameter σ.

**Figure 12:**
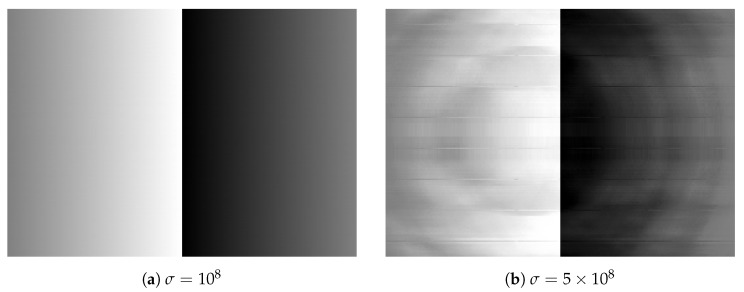
Histogram equalization by the proposed method with relatively large values of σ: (**a**) No artifact is generated with $\sigma =10^{8}$. (**b**) An artifact is generated with $\sigma =5\times 10^{8}$.

We investigate the limitation of the proposed method on the value of σ. Figure 12 shows the results of histogram equalization for the input image in Figure 8a, where Figure 12a,b correspond to $\sigma =10^{8}$ and $5\times 10^{8}$, respectively. Figure 12a is similar to Figure 9a. However, in Figure 12b, we observe an artifact. As a result, we estimate that the limitation of the value of σ exists between $10^{8}$ and $5\times 10^{8}$, and recommend using the value of σ smaller than $10^{8}$.

**Figure 13:**
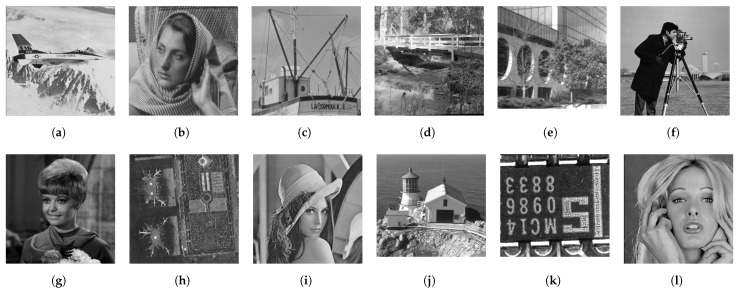
Grayscale images in the SIDBA image dataset [28]: (**a**) Airplane, (**b**) Barbara, (**c**) Boat, (**d**) Bridge, (**e**) Building, (**f**) Cameraman, (**g**) Girl, (**h**) Lax, (**i**) Lenna, (**j**) Lighthouse, (**k**) Text, (**l**) Woman.

**Figure 14:**
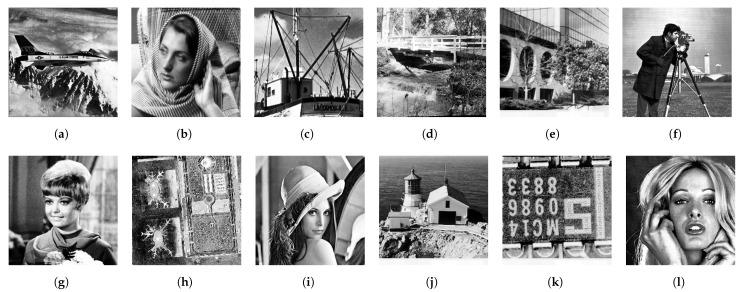
Nikolova and Steidl’s exact histogram equalization: (**a**) Airplane, (**b**) Barbara, (**c**) Boat, (**d**) Bridge, (**e**) Building, (**f**) Cameraman, (**g**) Girl, (**h**) Lax, (**i**) Lenna, (**j**) Lighthouse, (**k**) Text, (**l**) Woman.

Figure 13 shows twelve grayscale images in the Standard Image Data-BAse (SIDBA) [28], which are enhanced by Nikolova and Steidl’s exact histogram equalization as shown in Figure 14, where the contrast of all images is enhanced well because the histogram of each image is exactly equalized.

**Figure 15:**
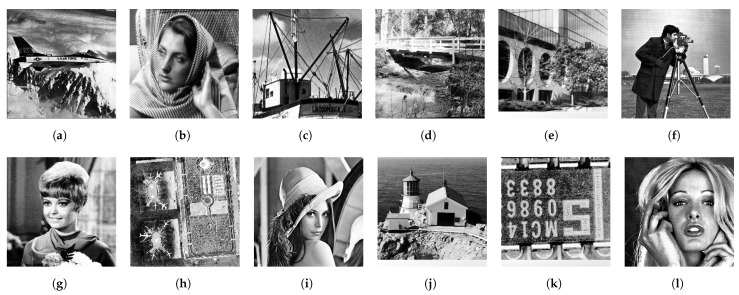
The proposed exact histogram equalization: (**a**) Airplane, (**b**) Barbara, (**c**) Boat, (**d**) Bridge, (**e**) Building, (**f**) Cameraman, (**g**) Girl, (**h**) Lax, (**i**) Lenna, (**j**) Lighthouse, (**k**) Text, (**l**) Woman.

Similarly, Figure 15 shows the results by the proposed method, which also equalizes the histogram of each image exactly. Therefore, the output images of both methods are similar to each other.

**Figure 16:**
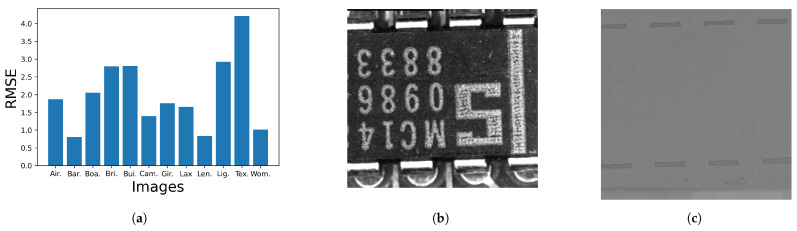
Comparison between Figure 14 and Figure 15: (**a**) RMSEs of twelve grayscale images; (**b**) The “Text” image obtains the largest RMSE value in (**a**); (**c**) Difference from the neutral gray indicates the difference between Nikolova and Steidl’s and the proposed methods. (**a**) Root mean squared error; (**b**) Text (identical to Figure 13k); (**c**) Difference image.

Figure 16a shows the root mean squared error (RMSE) between Nikolova and Steidl’s output image and the corresponding output image by the proposed method, where “Text” image shown in (**b**) get the largest RMSE value, but the difference is small as visualized in (**c**), where error-free pixels have the neutral gray value 127.

**Figure 17:**
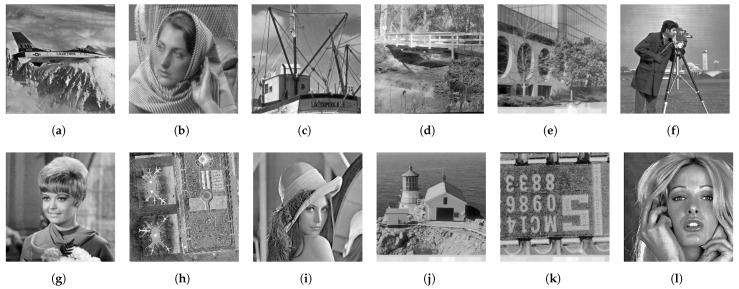
Nikolova and Steidl’s exact histogram specification: (**a**) Airplane, (**b**) Barbara, (**c**) Boat, (**d**) Bridge, (**e**) Building, (**f**) Cameraman, (**g**) Girl, (**h**) Lax, (**i**) Lenna, (**j**) Lighthouse, (**k**) Text, (**l**) Woman.

**Figure 18:**
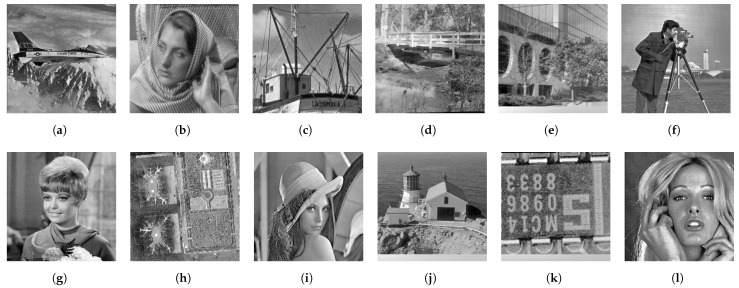
The proposed exact histogram specification: (**a**) Airplane, (**b**) Barbara, (**c**) Boat, (**d**) Bridge, (**e**) Building, (**f**) Cameraman, (**g**) Girl, (**h**) Lax, (**i**) Lenna, (**j**) Lighthouse, (**k**) Text, (**l**) Woman.

We also compare the results of exact histogram specification with the Gaussian target histogram, which are shown in Figure 17 and Figure 18 for Nikolova and Steidl’s and the proposed methods, respectively. In Figure 17, we observe that the obtained contrast is not so high as that of Figure 14 because the Gaussian target histogram suppresses the numbers of extremely dark and bright pixels, and assigns many pixel values to middle-range gray pixels. As a result, the dynamic range of gray pixels is boosted relatively. The output images in Figure 18 have a similar tendency to that in Figure 17 because the proposed method also obtains an exact Gaussian target histogram as well as Nikolova and Steidl’s method.

**Figure 19:**
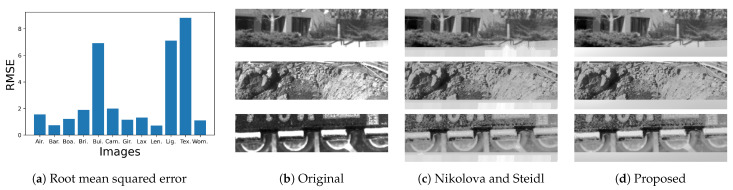
Comparison between Figure 17 and Figure 18: (**a**) RMSEs of twelve grayscale images; (**b**) The bottom areas of the original “Bui.”, “Lig.” and “Tex.” images are arranged from top to bottom; (**c**) the same parts of the results by Nikolova and Steidl’s method; (**d**) the same parts of the results by the proposed methods.

Figure 19a shows the RMSE between the corresponding output images in Figure 17 and Figure 18, where three images “Bui.”, “Lig.” and “Tex.” have relatively large RMSE values. We can find the differences at the bottom of those images as shown in Figure 19b, where the bottom areas of “Bui.”, “Lig.” and “Tex.” are arranged from top to bottom. The corresponding results by Nikolova and Steidl’s and the proposed methods are shown in Figure 19c and Figure 19d, respectively, where we can see artifacts in (**c**) similar to that in Figure 8b. On the other hand, Figure 19d has no such artifacts.

**Figure 20:**
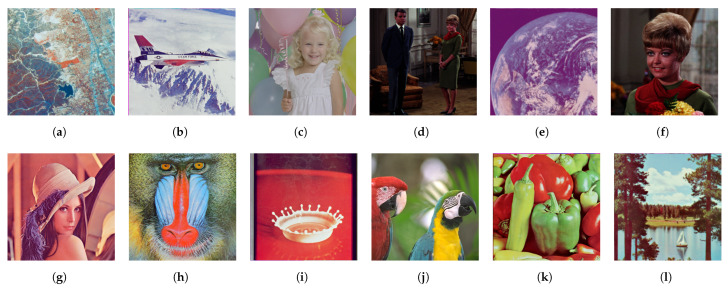
Color images in the SIDBA image dataset [28]: (**a**) Aerial, (**b**) Airplane, (**c**) Balloon, (**d**) Couple, (**e**) Earth, (**f**) Girl, (**g**) Lenna, (**h**) Mandrill, (**i**) Milkdrop, (**j**) Parrots, (**k**) Peppers, (**l**) Sailboat.

**Figure 21:**
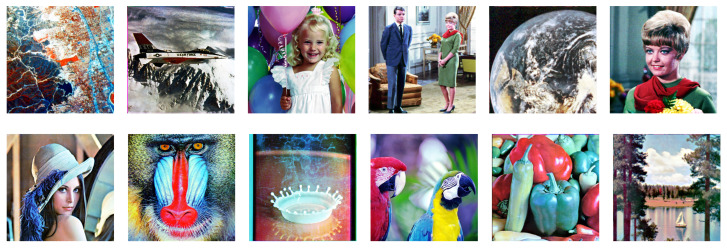
Results of separable histogram equalization with Nikolova and Steidl’s exact histogram equalization algorithm.

Figure 20 shows twelve color images in the SIDBA image dataset [28], which are enhanced by a separable histogram equalization that applies Nikolova and Steidl’s exact histogram equalization algorithm to each color channel separately as shown in Figure 21, where we can see that the contrast of every image is enhanced, but the hue has changed from the original images in Figure 20.

**Figure 22:**
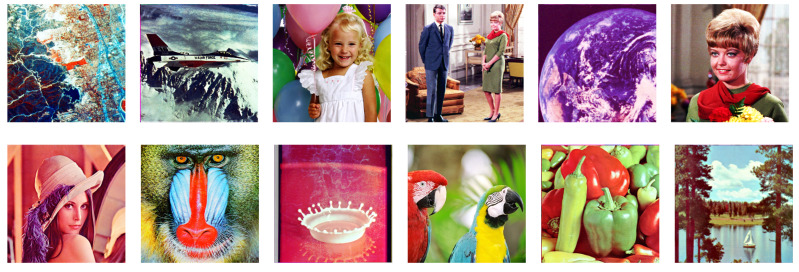
Results of the proposed exact histogram equalization.

Figure 22 shows the results from the proposed histogram equalization method described in Section 5.2, where we can see that the hue of each image is closer to the original one than that in Figure 21.

**Figure 23:**
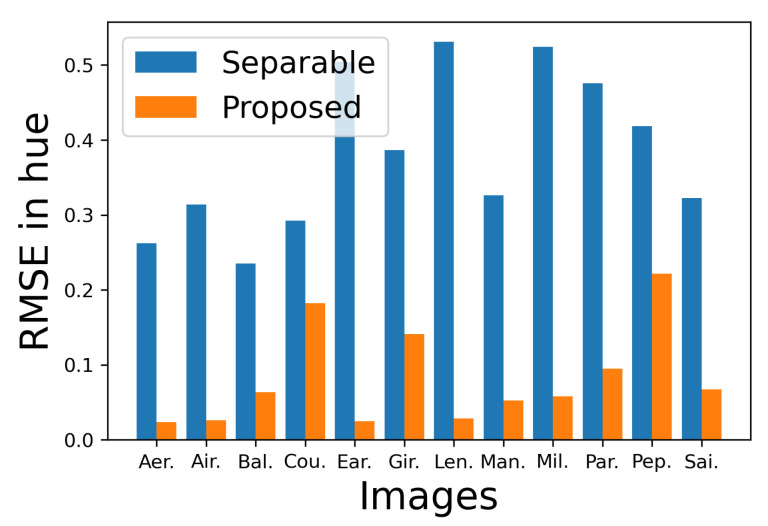
Comparison of the RMSE of hue of the images in Figure 21 and Figure 22 from the original images.

Figure 23 shows the RMSE of hue of each output image from the corresponding original image, where the vertical and horizontal axes denote the RMSE value and the images, respectively, and the blue and orange bars denote the separable and proposed methods, from which we observe that the proposed method preserves the original hue better than the separable method.

**Figure 24:**
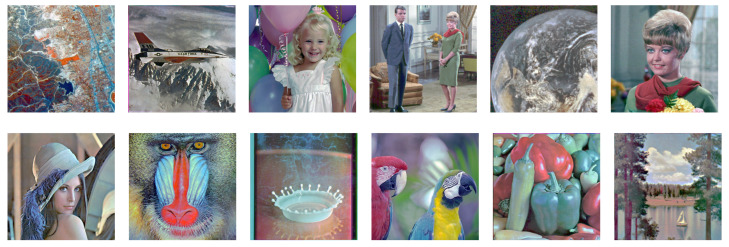
Results of the separable histogram specification with Nikolova and Steidl’s exact histogram specification algorithm.

Figure 24 shows the results of a separable histogram specification that applies Nikolova and Steidl’s exact histogram specification algorithm to each color channel separately. Similarly to the above results of histogram equalization in Figure 21, here we can see the hue change from the original images.

**Figure 25:**
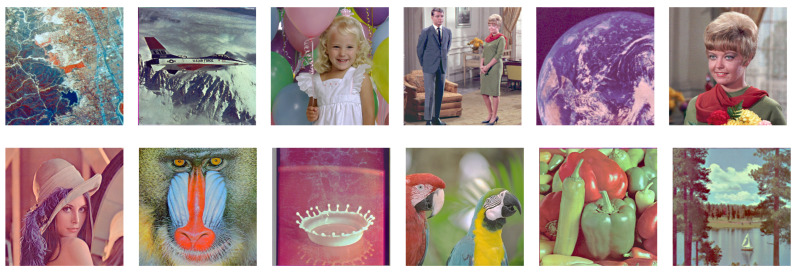
Results of the proposed exact histogram specification.

On the other hand, Figure 25 shows the results by the proposed histogram specification method, where we can also see that the hue of each image is closer to the original one than that in Figure 24.

**Figure 26:**
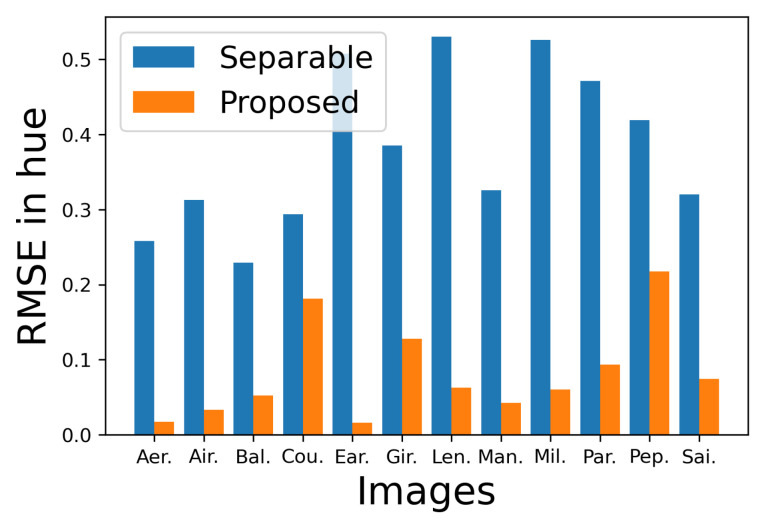
Comparison of the RMSE of hue of the images in Figure 24 and Figure 25 from the original images.

Figure 26 shows the RMSE of hue of each output image from the corresponding original image, from which we also observe that the proposed method preserves the original hue better than the separable method.

The above experimental results demonstrate the effectiveness of the proposed local contrast-based pixel ordering method for exact histogram equalization and specification compared with the state-of-the-art method by Nikolova and Steidl [13]. A drawback of Nikolova and Steidl’s algorithm observed in Figure 8b, where a stripe pattern occurred on flat areas, may be a newfound phenomenon. The proposed method can avoid the occurrence of such false patterns.

In the above experiments, although we used a Gaussian target histogram for histogram specification constantly, we can choose other candidates such as multimodal distributions without any problems.

For the implementation of Nikolova and Steidl’s algorithm described in Algorithm 1, we need to use sparse matrices even if the input image is not so large, e.g., the original image in Figure 1a with 256×256 pixels because the difference operator *G* in (7) becomes a very large matrix with $r\times mn\approx 8.56\times 10^{9}$ elements. On the other hand, the proposed method can be implemented without sparse matrices.

**Figure 27:**
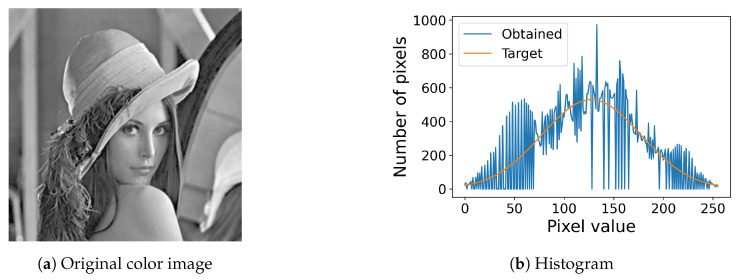
Histogram specification by Ramos’s algorithm [16]: (**a**) histogram specified image; (**b**) obtained and target histograms.

Additionally, we implemented Ramos’s recent algorithm for histogram specification [16]. Figure 27 shows the result of Ramos’s histogram specification, where Figure 27a shows the output image computed from the input image in Figure 1a with the Gaussian target histogram with mean 127.5 and standard deviation 50. Figure 27b shows the obtained histogram of the output image in Figure 27a in a blue line with the target histogram in the orange line. This figure shows that Ramos’s algorithm is not an exact histogram specification method, and the obtained histogram is similar to that of the conventional histogram specification shown in Figure 2b. On the other hand, the proposed method gives exactly the same histogram as a prescribed target histogram as shown in Figure 2c. In Ramos’s formulation of $l^{p}$ norm minimization, we set p=∞. For details, please refer to Ramos’s original paper [16]. For the implementation of Ramos’s algorithm, we need to use sparse matrices with about mn×L elements as well as Nikolova and Steidl’s one.

A limitation of the proposed method is that users need to prepare a target histogram for histogram specification. However, it may be difficult to know the optimal shape of the histogram for a given image in advance. The estimation of suitable target histograms for given images will be a subject to be considered in the future.

## 7. Conclusions

In this paper, we proposed a method for ordering pixels in an image for exact histogram specification, and compared it with the state-of-the-art algorithm by Nikolova and Steidl [13]. We divided the problem of pixel ordering into small subproblems which can be solved memory-efficiently. This idea makes the problem of exact histogram specification more tractable than ever. The proposed method uses the Gaussian filter without approximation instead of the nonlinear filter used in Nikolova and Steidl’s algorithm. We use the local contrast defined with the Gaussian-filtered image as a key to ordering pixels. As a result, it was experimentally demonstrated that we could avoid the occurrence of false patterns that were observed in the results obtained by Nikolova and Steidl’s algorithm.

We also extended the proposed method for grayscale images to that for color images. Experimental results showed that the proposed method keeps the hue of the original images better than conventional separable methods of histogram equalization and specification for color images.

Our future work will include the development of a method for making a preferable target histogram for histogram specification depending on a given input image.

## Data Availability

SIDBA, the Standard Image Data-BAse can be downloaded from http://www.ess.ic.kanagawa-it.ac.jp/app_images_j.html (accessed on 21 June 2022).

## Abbreviations

The following abbreviations are used in this manuscript:

LM	Local mean method
WA	Wavelet-based exact pixel ordering algorithm
TV	Total variation
SIDBA	The Standard Image Data-BAse
RMSE	Root mean squared error
